# Comparative genomics of the *Erwinia* and *Enterobacter* olive fly endosymbionts

**DOI:** 10.1038/s41598-018-33809-w

**Published:** 2018-10-29

**Authors:** Anne M. Estes, David J. Hearn, Sonia Agrawal, Elizabeth A. Pierson, Julie C. Dunning Hotopp

**Affiliations:** 1 Institute for Genome Sciences, University of Maryland School of Medicine, Baltimore, MD 21201 USA; 2 Department of Biological Sciences, Towson University, Baltimore, MD 21252 USA; 3 Department of Horticultural Sciences, Texas A & M University, College Station, TX 77843 USA; 4 Department of Microbiology and Immunology, University of Maryland School of Medicine, Baltimore, MD 21201 USA; 5 Present Address: Department of Biological Sciences, Towson University, Baltimore, MD 21252 USA

## Abstract

The pestivorous tephritid olive fly has long been known as a frequent host of the obligately host-associated bacterial endosymbiont, *Erwinia dacicola*, as well as other facultative endosymbionts. The genomes of *Erwinia dacicola* and *Enterobacter* sp. OLF, isolated from a California olive fly, encode the ability to supplement amino acids and vitamins missing from the olive fruit on which the larvae feed. The *Enterobacter* sp. OLF genome encodes both uricase and ureases, and the *Er*. *dacicola* genome encodes an allantoate transport pathway, suggesting that bird feces or recycling the fly’s waste products may be important sources of nitrogen. No homologs to known nitrogenases were identified in either bacterial genome, despite suggestions of their presence from experiments with antibiotic-treated flies. Comparisons between the olive fly endosymbionts and their free-living relatives revealed similar GC composition and genome size. The *Er*. *dacicola* genome has fewer genes for amino acid metabolism, cell motility, and carbohydrate transport and metabolism than free-living *Erwinia* spp. while having more genes for cell division, nucleotide metabolism and replication as well as mobile elements. A 6,696 bp potential lateral gene transfer composed primarily of amino acid synthesis and transport genes was identified that is also observed in *Pseudomonas savastanoii* pv *savastanoii*, the causative agent of olive knot disease.

## Introduction

Female tephritid flies cause extensive and costly crop damage by ovipositing their eggs into intact fruit still on the tree. The olive fly, *Bactrocera oleae*, is an unusual tephritid fly in that the female prefers to oviposit her eggs in unripe, green, intact olives that are protected by secondary chemicals, although larvae can develop in olive fruits of any degree of ripeness^1^. The olive fly has been difficult to manage due to its ability to develop resistance to pesticides, such as organophosphates, pyrethroids, and spinosads^2^. Sterile insect technique has been employed, whereby laboratory-diet-reared, reproductively-sterile, male flies are released to mate with wild females. It has had limited success for the olive fly^3^, due in part to the difficulty establishing and maintaining healthy, competitive olive fly laboratory colonies on an artificial diet.

Comparisons between diet-fed laboratory colonies and wild flies reveal different bacterial communities associated with the fly^3^. These native microbial communities may aid the wild fly in feeding on unripe olives, conferring resistance to pesticides, or otherwise enhancing the fly’s health. As such, an understanding of these microbes and their metabolic potential may help inform future studies on the olive fly microbiome and aid in managing this agricultural pest.

As early as 1909, one bacterial species, later identified as *Erwinia dacicola*^4^, was reported to be associated with wild olive flies as a specific endosymbiont^5^. *Er*. *dacicola* is widespread, found in >90% of the individuals within wild populations tested in the United States, Italy, Israel, Greece and Spain^6–9^.

When present, *Er*. *dacicola* may be found in all life stages, is most abundant in the mated, ovipositing female fly^6^, and appears to be obligately host-associated in that it cannot currently be cultured in the laboratory^4,10^. In larvae, *Er*. *dacicola* may entirely fill the midgut crypts, whereas in adults they fill an evagination off the foregut called the esophageal bulb and an evagination off the female hindgut at the ovipositor^10^.

In addition to *Er*. *dacicola*, several other bacterial taxa have occasionally been identified from different populations of olive flies as transiently associated bacteria^3^. Those taxa found most frequently in a given population include, *Enterobacter sp*. in United States fly populations (referred to as *Enterobacter* sp. OLF in this manuscript)^10^, *Pseudomonas putida* in Italian fly populations^8,11,12^, and *Acetobacter tropicalis* in Greek fly populations^9^. *Enterobacter sp.*^6,10^ and *A*. *tropicalis*^9^ have been found in low densities in all life stages of olive flies from some laboratory colonies, although they may be overlooked in wild populations, possibly due to the techniques being used for screening or the focus on *Er*. *dacicola*. *P*. *putida* is being investigated as a potential probiotic for olive fly colonies in Italy^12^. These non-*Erwinia* bacteria are thought to primarily be acquired from the environment during feeding^8^. Bacteria obtained from feeding would more likely be present in the gut lumen, but outside of the specialized foregut evagination where *Er*. *dacicola* resides. Faithful vertical transmission of *Enterobacter sp*., *A*. *tropicalis*, and *P*. *putida*, from a female to her offspring has not been specifically studied.

Due to the global economic impact of the olive fly, the decrease in fitness of antibiotic-treated flies^13,14^, and the correlation between different microbiomes and poorer health of laboratory flies reared for insect control, the olive fly appears to have a specific, beneficial microbiome composed of the endosymbiont *Er*. *dacicola* and perhaps other, more transient bacteria acquired during feeding that might be considered facultative endosymbionts. We sequenced the genomes of two bacterial endosymbionts, *Er*. *dacicola* and *Enterobacter* sp. OLF, isolated from wild California olive flies, in order to determine their functional potential. These bacteria were the focus of this study because of the near ubiquitous presence of *Er*. *dacicola* in wild flies^4,6,10^ and the presence of *Enterobacter* sp. OLF in wild olive fly populations in the United States and laboratory colonies^6,10^. Better understanding the basic biology of the fly-bacterial symbiosis, including the microbial genome and encoded metabolic potential, will inform future studies on this symbiosis and aid in the management of this agricultural pest.

## Results

### Sequencing and assembly

Paired end Illumina sequencing data were generated for *Enterobacter* sp. OLF from genomic DNA that was extracted from a pure culture of a single isolate. *Er*. *dacicola* is not currently culturable on standard media, including media on which plant pathogenic relatives, such as *Erwinia amylovora*, can be cultured^4,10^. However, esophageal bulbs are dominated by *Er*. *dacicola*^10^. Therefore, genomic DNA was extracted from bacteria that were isolated from four separate pools of esophageal bulbs from ~1-month-old surface-sterilized olive flies collected in Orville, CA, USA. One pool was discarded that was found to also contain *Enterobacter* DNA based on 16 S rRNA amplification and sequencing from each of the pools. Whole genome amplification was conducted on the remaining three esophageal bulbs and confirmed to be dominated by *Er*. *dacicola* by 16 S rRNA amplification and sequencing. All of the samples contained only the *Er*. *dacicola* htB genotype, consistent with previous sequencing of olive flies from the Southwestern United States^10^.

*De novo* assemblies were constructed from paired end Illumina data for both genomes using ABySS-pe v. 1.0.15^15^. *Enterobacter* sp. OLF and *Er*. *dacicola* are similar to their close relatives in terms of both %GC and genome size (Tables 1 and 2). We are confident that >98.5% of each these genomes was sequenced and assembled given the 300X sequencing depth and that the previously reported Illumina-based sequencing of the *Escherichia coli* genome to 50X sequencing depth and assembly in ABySS, yielded >98.5% of the genome^16^.

**Table 1:** Genome properties of *Er*. *dacicola* and *Enterobacter* sp. OLF.

Organism	Er. dacicola	Enterobacter sp. OLF
Size (bp)	2,860,970	5,068,785
Number of scaffolds	1,031	68
N50	5.48 kbp	180 kbp
Maximum scaffold size	79 kbp	362 kbp
G + C content (%)	52.2	55.1
Protein coding (%)	78.5	89.1
Coding sequences (CDS)	4,039	4,760
Average CDS size (bp)	563	949
rRNA operons	6	8
tRNAs	43	58

**Table 2:** Genomes, genome characteristics, and accession numbers of bacteria that were used for the comparative genomic analyses.

Organism	Pathogenic	Free-living	Size (Mbp)	GC	GenBank
*Enterobacter cloacae* subsp. *cloacae* ATCC 13047	Yes	Yes	5.59	54.6	CP001918
*Enterobacter cloacae* subsp. *dissolvens* SDM	Yes	Yes	4.97	55.1	CP000653
*Enterobacter* sp. 638	No	Yes	4.66	52.9	CP000653
*Enterobacter* sp. OLF	No	Yes	5.07	55.1	LJAN00000000
*Enterobacter aerogenes* EA1509E	No	Yes	5.59	54.9	FO203355
*Enterobacter asburiae* LF7a	No	Yes	5.01	53.8	CP003026
*Erwinia amylovora* ATCC 49946	Yes	No	3.91	53.6	FN666575
*Erwinia amylovora* CFBP1430	Yes	No	3.83	53.6	FN434113
*Erwinia dacicola*	No	No	2.87	52.3	LJAM00000000
*Erwinia pyrifoliae* DSM 12163	Yes	No	4.07	53.4	FN392235
*Erwinia pyrifoliae* Ep1/96	Yes	No	4.07	53.4	FP236842
*Erwinia* Ejp617	Yes	No	3.96	53.6	CP002124
*Erwinia billingiae*	No	Yes	5.37	55	FP236826
*Erwinia tasmaniensis* Et1/99	No	Yes	4.07	53.4	CU468135

### Provenance of *Er*. *dacicola* scaffolds

Given that *Er*. *dacicola* DNA could not be obtained from pure culture, we first sought to establish the provenance of the scaffolds sequenced. The scaffolds were searched using BLASTN against the olive fly mitochondrial genome and the two bacterial genomes sequenced on other lanes on the same sequencing run—the *Enterobacter* sp. OLF genome and a *Klebsiella* genome. Five scaffolds were identified as being of mitochondrial origin and were excluded from subsequent analyses. Three scaffolds were identified with >90% identity across >90% of the length of the scaffold, to the *Klebsiella* and *Enterobacter* sp. OLF genomes. However, in all cases the matches were to rRNA that is highly homologous between these taxa. Given that no other matches were identified with >90% identity across >90% of the scaffold, we are confident that there was not cross-contamination between the sequencing lanes or projects.

Recently, a separate draft genome assembly was generated for *Er*. *dacicola* from 8 single-cell and 2 metagenome libraries constructed from olive flies collected in Greece^17^, which will be referred to as the Liverpool assembly. The Liverpool assembly has a similar GC content (53.5%) to our assembly (referred to as the US assembly), but the Liverpool assembly has a reported genome size (2.1 Mbp), which is substantially less than the 2.9 Mbp US assembly. To compare the two data sets, the US reads and the single run of Liverpool reads with the greatest sequencing depth were aligned to the larger US genome and the sequencing depth was calculated. For US reads, the sequencing depth distribution across these scaffolds was unimodal, but asymmetric, with a sequencing depth mean of 645X, median of 476X, and mode of 280X (Supplementary Fig. S1). The mode is typically the most reliable metric for sequencing depth, thus the actual sequencing depth is likely 280X but there are large portions of the genome that are over-represented (Supplementary Fig. S1). Such over-representation is common in sequencing projects and may be due to the whole genome amplification, sequencing, and/or assembly (e.g. the presence of collapsed repeats in the assembly). Collapsed repeats are expected in the assembly given that only short reads were generated from a paired end library with a relative short insert size. By comparison, for the Liverpool reads, the sequencing depth distribution across these scaffolds was largely unimodal and symmetric, with a sequencing depth mean of 107X, median of 102X, and a mode of 109X (Supplementary Fig. S1). A visual examination of the frequency distribution of the sequencing depth of the Liverpool data reveals five local modes (Supplementary Fig. S1), at 2x, 50x, 200x, 400x, and 700x. Those scaffolds with abnormally high coverage may be collapsed repeats in the assembly from genome duplications or on plasmids with a higher copy number than the genome (Supplementary Data File S1). As such, these genes will likely be of interest in future studies on *Er*. *dacicola*. An examination of the regions with lower than average sequencing depth revealed a possible similar association with mobile elements, such as phage (Supplementary Data File S1).

### *Erwinia dacicola* comparisons

We sought to compare the genome annotation of the US and Liverpool assemblies using Jaccard-ortholog clusters (JOCs) in SYBIL^18^, after the Liverpool assembly was re-annotated with the IGS pipeline to facilitate these comparisons. A publicly available transcriptome assembly was also included, which was generated from sequence data from two libraries constructed from rRNA-depleted RNA isolated from pools of olive fly larvae collected on green and black olives in Israel^19^, referred to as the Mediterranean assembly. It is important to remember that a transcriptome assembly is not expected to recover all of the genes in a genome, but merely the genes that are transcribed under the conditions tested, in this case green or black olives as a larval food source. However, at between 2.70 and 2.77 Mbp, it is more similar in size to the US assembly than the Liverpool assembly.

**Figure 1:**
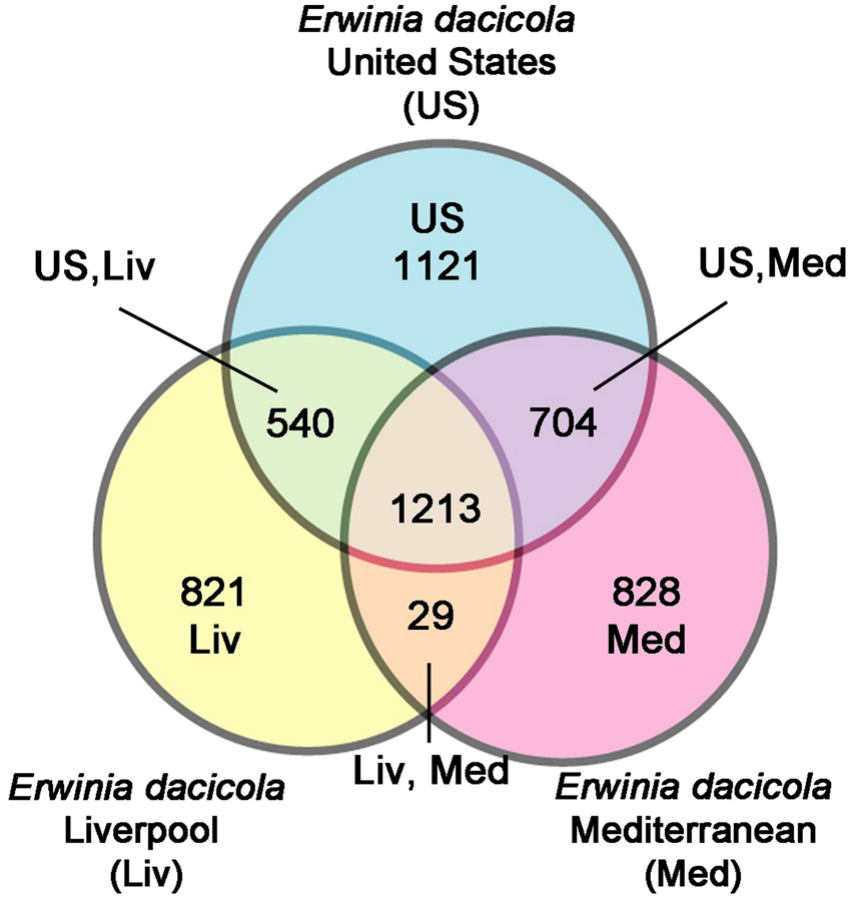
Venn Diagram of *Er*. *dacicola* genomes and transcriptome. A Venn diagram was constructed using SYBIL-generated Jaccard-ortholog clusters (JOCs)^18^ for the *Er*. *dacicola* genomes generated in the US and Liverpool as well as the transcriptome generated in Israel, after the Liverpool assembly was re-annotated with the IGS pipeline to facilitate these comparisons.

There were 1,213 JOCs that were shared between all three assemblies (Fig. 1). In all three assemblies, there were 821-1,121 genes that did not cluster into JOCs (Fig. 1). Surprisingly, given its smaller size, the Liverpool assembly maintained a similar number of genes that did not cluster into JOCs relative to the Mediterranean and US assemblies (Fig. 1). Some of these assembly-specific genes reflect annotation differences. For instance, 170 small hypothetical proteins (<60 aa) were shared between the US and Liverpool assemblies, which were both annotated with the IGS annotation pipeline. There are 540 JOCs shared between the US and Liverpool assemblies but not found in the Mediterranean assembly, which could reflect genes not transcribed in larvae feeding on green and black olives. There are 704 JOCs shared between the US and Mediterranean assemblies but not found in the Liverpool assembly (Fig. 1). These include 4 ribosomal proteins and 3 DNA polymerase III subunits, which are likely essential, as well as 3 DNA polymerase V subunits, 6 proteins from the type IV secretion system, at least 8 proteins involved in cell division, and 2 of the urease accessory proteins. There are also at least 28 phage genes and at least 20 conjugal transfer proteins found in the US and Mediterranean assemblies, but not the Liverpool assembly. This is likely due to the smaller size of the Liverpool assembly, which in turn is due to differences in the methods employed to remove contaminating scaffolds. While both methods are valid, they highlight a philosophical difference between balancing contaminant removal with retaining bona fide genomic sequences that lack a strong signal of vertical inheritance. Only 29 JOCs were identified as shared only between the Liverpool and Mediterranean assemblies and not in the US assembly, of which over half are hypothetical proteins. Taken together, these results indicate that the US assembly is the most comprehensive assembly of *Er*. *dacicola*.

### Biosynthesis of Amino Acids, Vitamins, and Cofactors

One of our hypotheses is that the endosymbionts in the olive fly provide amino acids, vitamins, and cofactors to their insect hosts to supplement components missing from the host diet, as reported for numerous other insect endosymbionts^20^. Olive fruits, especially unripe, green fruits, lack several amino acids that are essential for basic metabolic needs of the insect^21–24^. The genome of *Enterobacter* sp. OLF encodes multiple different amino acid biosynthesis pathways, suggesting that it might be able to survive outside of the insect without amino acid supplementation. In contrast, the *Er*. *dacicola* genome only encodes pathways for glutamate, glutamine, glycine, isoleucine, and proline synthesis. Additionally, partial pathways are present for phenylalanine and tryptophan biosynthesis, isoleucine biosynthesis, and arginine/ornithine biosynthesis. Given that the genomes are not closed/complete, as well as our incomplete understanding of alternate metabolic pathways and enzymes in bacteria, it is not possible to say whether the pathways are functional. Both the *Er*. *dacicola* genome and *Enterobacter* sp. OLF genome have genes for biotin, flavin, folate, thiamin, heme, coenzyme A, ubiquinone, molybdopterin, lipoate, menaquinone, and molybedenum cofactor biosynthesis.

### Degradation pathways

Olives are rich in lipids and are known for the oils they produce. The *Enterobacter* sp. OLF genome has the genes for fatty acid degradation, oleate β-oxidation, and glycerol degradation. However, the genes for lipid degradation are not detected in the *Er*. *dacicola* genome (Supplementary Table S1).

Olive fruits are chemically defended with multiple phenolic acids and other secondary metabolites, perhaps to decrease predation^25^. Oleuropein, the most abundant phenol in unripe olives, is known to be degraded by β–D glucosidase encoded by *blgC* and esterases^26^. The *Enterobacter* sp. OLF genome lacks *bglC*. However, it has pathways for degrading the aromatic compounds gallic acid, rutin, phenylacetate, phenylethylamine, 3-phenylpropanoate and 3-(3-hydroxyphenyl) propanoate, cinnamate and 3-hydroxycinnamate (Supplementary Table S2 and Supplementary Table S3). Gallic acid and rutin are known to be present in olives^27^.

Degradation of olive secondary metabolites has been considered as one potential role of *Er*. *dacicola*. The genomes of *Er*. *dacicola* and a related “soft-rot *Erwinia*” *Dickeya chrysanthemi* both have *bglC*, but this gene is lacking from other plant pathogenic and non-phytopathogenic *Erwinia* genomes. All of the *Erwinia* genomes examined (Table 2) have esterases, although the *Er*. *dacicola* genome has at least 6 esterases that were not found in other *Erwinia* genomes examined (Supplementary Table S1). Some esterases are known to degrade phenols, although this is not their sole purpose.

*Enterobacter* sp. OLF has the genetic potential to degrade a variety of nutritional compounds including glycogen, muropeptide, xyloglucan, urea, and starch. With the exception of proline degradation, the *Enterobacter* sp. OLF genome has multiple, complete pathways for degrading all twenty amino acids and taurine.

**Figure 2:**
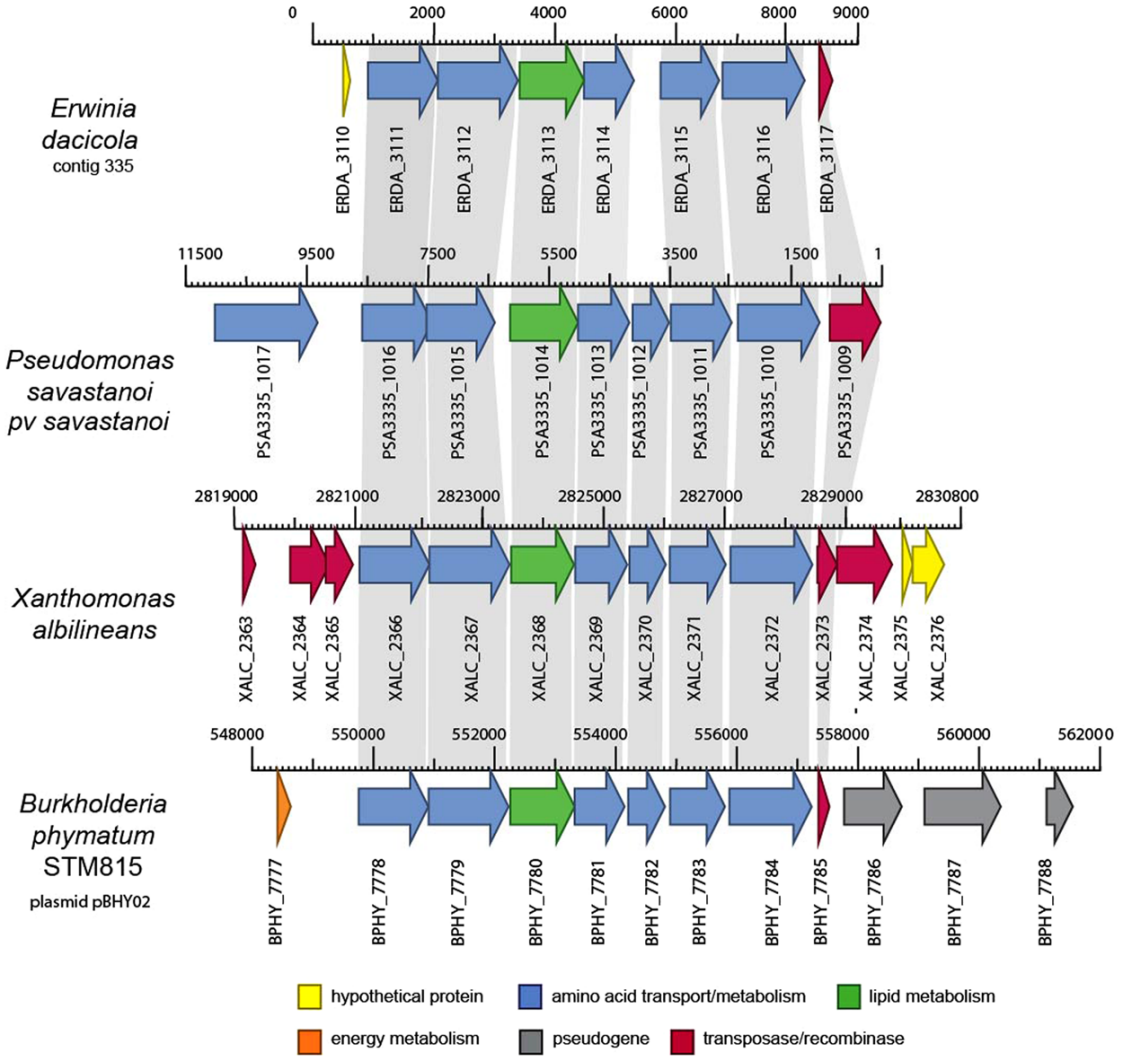
Lateral gene transfer. An alignment of the potential lateral gene transfer present is illustrated for *Er*. *dacicola* (LJAM00000000), *Pseudomonas savastanoii* pv. savastanoii NCPPB 3335 (GCA_000164015.2), *Xanthomonas albilineans* GPEPC73 (GCA_000087965.1), and plasmid pBPHY02 of *Burkholderia phymatum* STM815 (GCA_000020045.1). Homologous genes are connected with gray and were identified through best BLASTN matches. These genes are as follows from left to right (pyruvate aminotransferase (GED_3111), O-acetylhomoserine sulfhydrylase (GED_3112), fatty acid desaturase (GED_3113), family C26 peptidase (GED_3114), homoserine O-succinyltransferase (GED_3115), and a glutamine synthetase family protein (GED_3116)).

The *Er*. *dacicola* genome has a more limited set of amino acid degradation pathways including those for asparagine, aspartate, glutamic acid, glutamine, glycine, serine, threonine, arginine, and tryptophan. Only aspartate and arginine have multiple degradation pathways whereas a single pathway was identified for the other amino acids. All of these amino acids are found in olives, with aspartate and arginine together composing over 24% of the total amino acids in the olive^22,28^. Some of the genes for these pathways may have resulted from lateral gene transfer of a 6,696 bp region homologous to regions in *Pseudomonas savastanoi* pv. *savastanoi*, *Xanthomonas albilineans*, and a plasmid found in *Burkholderia phymatum* STM815 (Fig. 2). This gene region has the closest BLASTP match to *Pseudomonas savastanoi* pv. *savastanoi*, the causative agent of olive knot disease, is flanked by two transposases, and encodes a glutamine synthetase catalytic domain protein, a homoserine O-succinyltransferase for methionine biosynthesis, glutamine amidotransferase, fatty acid desaturase, O-acetylhomoserine sulfhydrylase, and pyruvate aminotransferase (Fig. 2).

### Precursor metabolites and energy

The *Enterobacter* sp. OLF genome encodes numerous pathways for obtaining energy. Both aerobic and anaerobic respiration pathways are present. The genome also has genes for acetoin biosynthesis, the Entner-Doudoroff pathway, formate oxidation, glycolysis, gluconeogenesis, the glyoxylate cycle, hydrogen production, mixed acid fermentation, the pentose phosphate pathway, several pathways for pyruvate and succinate fermentation, and pathways for degrading a wide variety of carbohydrates.

The *Er*. *dacicola* genome has a subset of those energy obtaining pathways, but lacks the glyoxylate cycle and fermentation. For carbohydrate degradation, the *Er*. *dacicola* genome only encodes enzymes for using fructose, glucose and glucose-1-phosphate, mannitol, sucrose, lactose, sorbitol, trehalose, and myo-inositol.

### Nitrogen metabolism

Throughout its life cycle, the olive fly feeds on a diet low in free nitrogen. Experiments with antibiotic-treated olive flies have suggested that endosymbionts may fix nitrogen^14^. The *Enterobacter* sp. OLF genome encodes nitrogen regulation proteins *ntrC*, *nac*, *glnB*, while the *Er*. *dacicola* genome encodes nitrogen regulation proteins *glnBDL*, *ntrC*, and *NR*. Nitrite transporters and reductases are also found in the *Enterobacter* sp. OLF genome. The proteins for degrading urea via urease are present in the *Enterobacter* sp. OLF genome, while the *Er*. *dacicola* genome only had two genes with homology to urease accessory proteins, UreG and UreF. The genes usually encoding nitrogenase activity were not found in the *Enterobacter* sp. OLF or the *Er*. *dacicola* genomes, although it is important to consider that these are draft genomes that are not closed/complete and as such the nitrogenase genes could be in gaps. However, we consider this highly unlikely.

### Surface structure

Lipopolysaccharide (LPS) and peptidoglycan are essential for bacterial outer membrane stability. LPS is composed of the lipid A hydrophobic anchor, a core oligosaccharide, and the O-antigen for host specificity. Changes in lipid A alter outer membrane permeability leading to changes in the ability of bacteria to withstand harsh environments or evade host detection^29^, as is seen in *Wolbachia* and *Borrelia burgdorferi*a that are missing the *lpx* genes^30^. In contrast, enteric, commensal bacteria of humans decorate their cell surface with hexa-acylated lipid A, which seems to aid the host in recognizing them as non-pathogenic^29^.

Both the *Enterobacter* sp. OLF and *Er*. *dacicola* genomes have the *lpx* genes for the lipid IV_A_ biosynthesis pathway and the *htrB* and *msbB* genes for (KDO)2-lipid A biosynthesis I, peptidoglycan, phospholipids, and cardiolipin. However, only two genes, *rfaC* and *rfaF*, of the lipid A-core biosynthesis pathway are identified in both genomes. Thus, neither *Er*. *dacicola* nor *Enterobacter* sp. OLF have the typical genes for synthesizing the outer oligosaccharide core or for complete synthesis of the inner oligosaccharide core. While *Enterobacter* sp. OLF has genes for the O7 antigen subunit of the LPS that is often associated with virulence, the genome of *Er*. *dacicola* lacks O7 antigen subunit genes.

### Flagellar genes

The *Enterobacter* sp. OLF genome contained all of the genes for flagellar biosynthesis. In contrast, the *Er*. *dacicola* genome has *flnA* and the flagellar biosynthesis protein, but is missing the majority of the flagellar genes. All other plant pathogenic and non-phytopathogenic *Erwinia* genomes examined encode a complete set of flagellar genes, suggesting that *Er*. *dacicola* has lost its flagella.

### COG clusters

**Figure 3:**
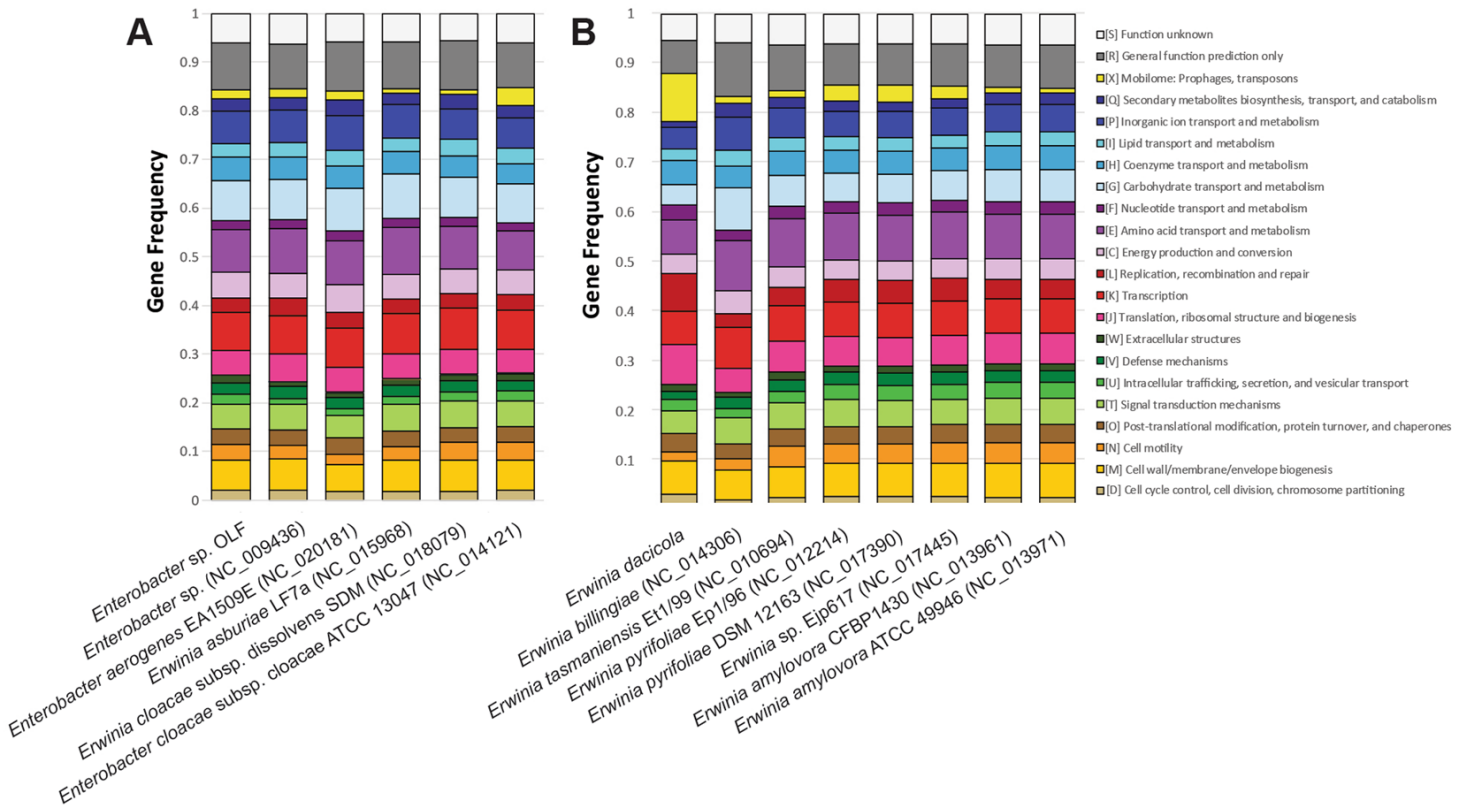
COG clusters for the different *Enterobacter* isolates. Frequencies of genes within COG categories are illustrate for the sequenced genomes of (**A**) free-living *Enterobacter* spp. relative to *Enterobacter* sp. OLF and (**B**) free-living *Erwinia* spp. relative to the *Erwinia dacicola* genome as defined in Table 2. Intracellular trafficking, secretion, and vesicular transport (COG U) and extracellular structures (COG W) are enriched between the free-living *Enterobacter* species and *Enterobacter* sp. OLF. All other categories are similar. Secondary metabolites biosynthesis, transport and catabolism (Q) COG is reduced when *Enterobacter* sp. OLF is compared to pathogenic *Enterobacter* spp. Carbohydrate transport and metabolism (COG G), cell motility (N), amino acid metabolism (E), and signal transduction mechanisms (T) are some COG categories decreased in the *Erwinia dacicola* genome versus the free-living *Erwinia* to which it was compared. Increased levels of prophages and transposons (X), replication, recombination, and repair (L), translation (J), cell division and chromosome partitioning (D), and nucleotide metabolism and transport (F) were identified in *Er*. *dacicola* relative to all other *Erwinia* spp.

The abundances of genes in COG categories in the *Enterobacter* sp. OLF genome are marginally different from all the examined pathogenic and non-pathogenic *Enterobacter spp* combined (χ^2^ = 36.5, df = 23, p-value = 0.04) (Table 2, Fig. 3). However, more significant differences are observed when the *Enterobacter* sp. OLF genome is compared to the pathogeneic and non-pathogenic susbsets separately. The genomes of *Enterobacter* sp. OLF and the *Enterobacter* species examined are enriched with genes belonging to two COG categories: intracellular trafficking, secretion, and vesicular transport (U) and extracellular structures (W), while *Enterobacter* sp. OLF and the non-pathogenic *Enterobacter spp*. genomes examined are enriched with prophages and transposons (X). In contrast, the genomes of *Enterobacter* sp. OLF and the *Enterobacter* species examined are depleted in signal transduction mechanisms (T) (χ^2^ = 100.14, df = 23, p-value < 1 × 10^−11^), whereas secondary metabolite biosynthesis, transport and catabolism (Q) COG is reduced when *Enterobacter* sp. OLF is compared to pathogenic *Enterobacter spp* (χ^2^ = 31.5, df = 23, p-value = 0.11).

The abundances of genes in *Er*. *dacicola* are significantly different from all of the other examined *Erwinia spp*. combined ((χ^2^ = 375.16, df = 22, p-value < 1 × 10^−10^), non-phytopathogens (χ^2^ = 779.0, df = 22, p-value = 1 × 10^−13^), and phytopathogens (χ^2^ = 276.0, df = 22, p-value = 1 × 10^−11^) (Table 2, Fig. 3). *Er*. *dacicola* has fewer genes in COG categories for carbohydrate transport and metabolism (G), cell motility (N), amino acid metabolism (E), secondary structure (Q), general functional prediction (R), and signal transduction mechanisms (T) than the free-living *Erwinia* to which it was compared. A corresponding increase of *Er*. *dacicola* genes are found for prophages and transposons (X), replication, recombination, and repair (L), translation (J), cell division and chromosome partitioning (D), and nucleotide metabolism and transport (F) (Fig. 3). Differences between *Er*. *dacicola* and the phytopathogenic *Erwinia spp*. include more genes in COGs X, D, and L, and fewer genes in cell motility (N).

### Comparisons with free living relatives

**Figure 4:**
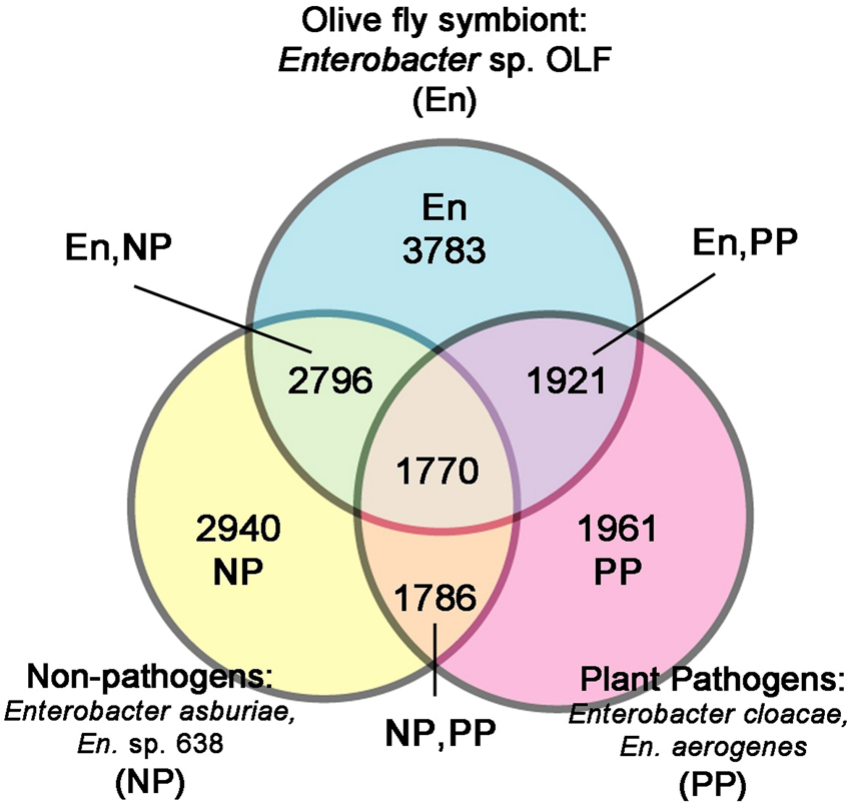
Venn Diagram *Enterobacter* sp. OLF. The shared core clusters of non-duplicated genes as determined by MUGSY are compared between *Enterobacter* sp. OLF, non-pathogenic *Enterobacter*, and pathogenic *Enterobacter* as defined in Table 2.

**Figure 5:**
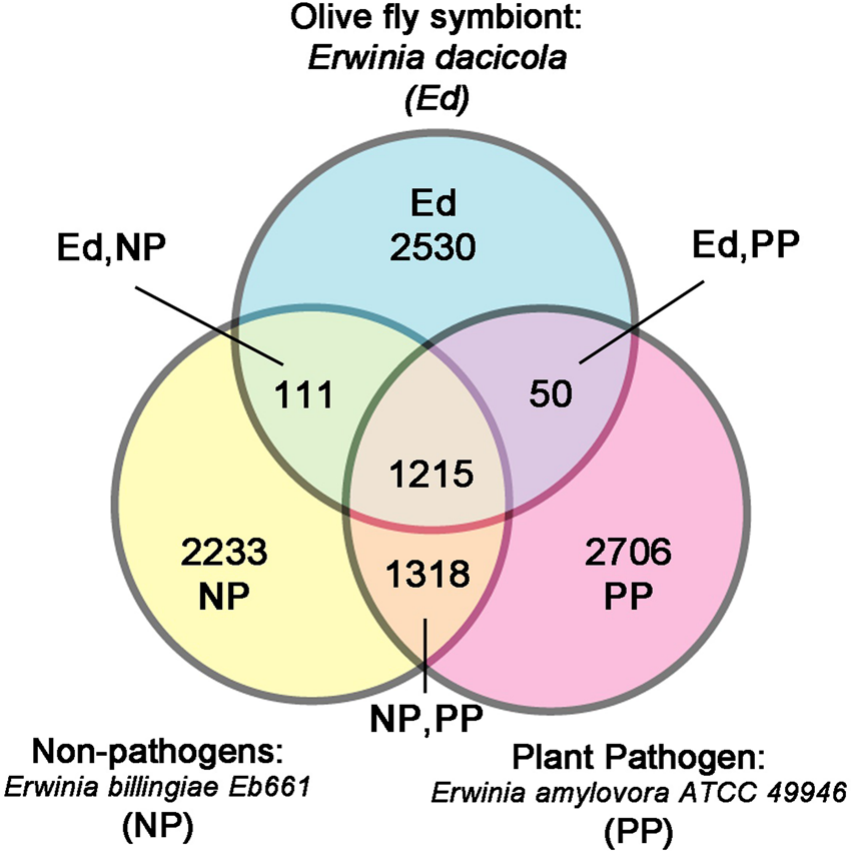
Venn Diagram *Er*. *dacicola*. The JOCs of non-duplicated genes as determined by SYBIL are compared between the olive fly endosymbiont, non-phytopathogenic *Erwinia*, and plant pathogenic *Erwinia*. *Er*. *dacicola* has approximately as many unique genes as the number of genes it shares with plant pathogens as defined in Table 2.

Using MUGSY annotator^31^, *Enterobacter* sp. OLF shares 2,796 core gene clusters with non-pathogenic relatives (*En*. *asburiae* and *Enterobacter sp*. *638*) while sharing 1,921 clusters with pathogenic relatives (*En*. *cloacae* and *En*. *aerogenes*) (Fig. 4, Supplementary Table S2). The genome of *Er*. *dacicola* was compared to the genomes of 2 representative, free-living *Erwinia* species, specifically the plant pathogen *Er*. *amylovora* ATCC 49946 and the non-phytopathogen *Er*. *billingiae* Eb661 using JOCs in SYBIL. There are 1,215 JOCs shared between all three species and 2,233-2,706 genes unique to each species (Fig. 5, Supplementary Table S1). While 1,318 JOCs were shared between the plant pathogen and the non-phytopathogen, only 50 JOCs were shared between the olive fly symbiont and the plant pathogen and 111 JOCs were shared between the non-phytopathogen and the olive fly symbiont. Among the genes absent from the *Er*. *dacicola* genome relative to the genomes of the other *Erwinia* spp. are a multitude of transporters, 8 chemotaxis genes, and 35 flagellar genes. The absence of these genes may be a result of the olive fly symbiont’s specialization, only living in the insect with vertical transmission. However, it is important to remember that there may be annotation differences that affect these clusters as well as genes missing in both the *Enterobacter* sp. OLF and *Er*. *dacicola* draft genomes.

### Unique genes

Pathway tools identified many more pathways present in the genome of *Enterobacter* sp. OLF than *Er*. *dacicola* (Supplementary Table S3). Some of the most interesting pathways included those for cellulose and chitin degradation, several hormone biosynthesis pathways (*cis*-zeatin, jasmonic acid, leukotriene), siderophore biosynthesis, and streptomycin biosynthesis. Pathways identified in the genome of *Er*. *dacicola*, but not *Enterobacter* sp. OLF included those for degrading alginate, myo-inositol, acetoacetate, and catechol as well as phosphatidylcholine resynthesis. Biosynthesis pathways for NAD salvage and chlorophyllide A biosynthesis are found only in the *Er*. *dacicola* genome.

## Discussion

### Genome characteristics of olive fly endosymbionts

In general, endosymbionts genomes are typically thought to be reduced in size with a highly skewed GC/AT content compared to those of free-living relatives^32,33^. As such, genome reduction and skewed nucleotide content might be expected in these endosymbiont genomes, particularly *Er*. *dacicola*, which is frequently associated with the olive fly and is obligately host-associated. However, the size and GC/AT content of both the *Enterobacter* sp. OLF and *Er*. *dacicola* genomes were similar to the genomes of the respective free-living relatives to which they were compared. This may be due, in part, to the fact that the olive fly is a holometabolous insect with a diet that varies during insect development. Highly reduced genomes are often associated with endosymbionts of hemimetabolous insect hosts where the bacterial endosymbionts are thought to supplement the host’s nutritionally unbalanced and monophagous diet^20^. In contrast, the olive fly feeds exclusively on olives, preferably unripe olives, during its larval stage, but becomes a generalist feeder on nectar, pollen, bird feces, and bacteria found on plant surfaces during its adult stage^8,34,35^. Thus, a co-evolved endosymbiont of a holometabolous insect may need a larger genetic repertoire to survive through the various life stages and diet changes. Alternatively, lack of genome reduction and nucleotide skew may be related to the age of the symbiosis. It has been estimated that the olive fly has associated with the olive tree for only ~50 million years^36^, and thus perhaps there has been insufficient time for the *Er*. *dacicola* genome to reduce in size and develop an altered GC skew. Despite the lack of clear evidence for genomic degradation, *Er*. *dacicola* was found to have fewer genes related to carbohydrate and amino acid transport and metabolism and a nearly complete loss of genes for cell motility compared to the free-living *Erwinia*, suggesting some genomic specialization has occurred.

### Nutritional – Amino acids

One hypothesis of our genomic study was that the olive fly endosymbionts may provide only those essential amino acids missing from the insect host’s diet, as reported for numerous other insect endosymbionts^20,37^. In contrast the combined genomes of *Er*. *dacicola* and *Enterobacter* sp. OLF have at least one pathway to synthesize all amino acids, essential and non-essential, regardless of the concentration and presence of the amino acid in olives. Experimental results suggest that perhaps diet supplementation by the microbiome is important in the adult stage where the fly is a generalist forager and the female needs protein for egg production^13,14,38^. The genomes of *Er*. *dacicola* and *Enterobacter* sp. OLF suggest that either one, or both, of these bacteria could supplement the amino acids in the fly’s diet. Since *Enterobacter* sp. OLF has multiple pathways for synthesizing each amino acid and it can be cultured, while *Er*. *dacicola* cannot, *Enterobacter* sp. OLF could potentially be added to artificial diets of reared olive flies.

All of the amino acid biosynthesis genes found in the *Er*. *dacicola* genome, are homologous to the genes of free-living *Erwinia* relatives, with the exception of genes that are similar to those from the olive knot pathogen *Ps*. *savastanoi* pv *savastanoi* located next to each other on a scaffold between two transposable elements (Fig. 2). This was not an area of atypical nucleotide content, suggesting that if this is a lateral gene transfer, it is not recent, or is from an organism with a similar di-nucleotide composition. Such lateral gene exchange is rarely seen in exclusively intracellular, obligate endosymbionts such as *Buchnera* in aphids or *Camponotus* in ants^33^. However, given the olive fly’s habit of feeding on bacteria from the olive tree phylloplane^8^, the extracellular lifestyle of the endosymbionts during the adult stage, and the examples of transfer of bacterial genes within the guts of other insects^39,40^, it is not surprising that amino acid genes missing from the *Er*. *dacicola* genome may have been acquired via lateral gene transfer, potentially from the olive knot pathogen in the olive fly gut.

### Nutritional - Carbohydrates

Olives change in chemistry during ripening. Sugars predominantly found in unripe olives are glucose, sucrose, mannitol, and inositol, while in ripe olives, fructose and mannitol are most prevalent^41^. The *Er*. *dacicola* genome has the complete set of genes for glycolysis and the TCA cycle. In addition, it has enzymes for degrading fructose, glucose and glucose-1-phosphate, mannitol, sucrose, lactose, sorbitol, trehalose, and inositol, many of which are found in olives. However, overall, the *Er*. *dacicola* genome has fewer carbohydrate metabolism genes than its free-living relatives. One hypothesis is that the carbohydrate pathways in the *Er*. *dacicola* genome have degraded to reflect the olive’s carbohydrate composition, although adult flies will feed on the carbohydrate-rich honeydew excreted by sap feeding insects, which may have a different set of carbohydrates^35^.

### Nutritional - Vitamins

Most insects cannot synthesize thiamine, riboflavin, nicotinic acid, pyridoxine, pantothenic acid, folic acid, and biotin and thus need to obtain these vitamins from either their diet or endosymbionts. Yet the only vitamin present in high abundance in olive fruits is vitamin E^42^. Endosymbionts of blood-feeding^43^ and other insects^44,45^ provide vitamins missing from the insect’s diet. The genomes of both *Enterobacter* sp. OLF and *E*. *dacicola* have the potential to synthesize six of the seven vitamins essential for insect development. The *Er*. *dacicola* genome also has genes for synthesizing nicotinic acid via the NAD salvage pathway. The NAD salvage pathway is one of the handful of pathways that is found only in the *Er*. *dacicola* genome and not the *Enterobacter* sp. OLF genome.

### Nutritional – Nitrogen

Perhaps the olive fly diet is best compared to that of omnivorous ants and cockroaches that feed on nitrogen-limited diets. In these insect omnivores, endosymbionts supplement the host with nitrogen, sulfur, and lipids^46^. Antibiotic-treated olive fly females supplied with either only sugar, or sugar and urea, produced significantly fewer eggs than untreated flies that were fed diets of sugar and urea, or sugar with bird feces^14^. Thus olive fly endosymbiont genomes have been hypothesized to have nitrogen fixation or urease genes to generate ammonia, which may be used for synthesizing the amino acid glutamine in the olive fly^13,14^. The microbiome of other tephritid fruit flies has been found to have nitrogenase activity^38,47,48^ and uricase activity^49^. However, no homologs to known nitrogenase genes were found in the genome of either olive fly endosymbiont. The *Enterobacter* sp. OLF genome has a uricase gene and all of the canonical urease genes, while the *Er*. *dacicola* genome has an allantoate transport pathway. These findings suggest that nitrogen is acquired from urea, uric acid, allantoin, allanoic acid, and/or ammonia found in the bird feces that compose the adult fly’s diet or from recycling the olive fly waste products.

### Other non-nutritional benefits

Since olive flies are one of the few insects to feed on chemically defended, unripe olive fruit, it has been hypothesized that the endosymbionts may degrade the olive phenolics and other secondary metabolites to aid larval survival^10,14^. The presence in the *Enterobacter* sp. OLF genome of genes for degrading gallic acid and rutin, two secondary metabolites found in olives, suggests that *Enterobacter* sp. OLF could be important to larval olive fly survival in unripe olives.

Oleuropein is another of the dominant olive fruit phenolics thought to chemically protect the olive fruit flesh from insect pests as well as microbial pathogens. One oleuropein degrading gene, *bglC*, was found in the *Er*. *dacicola* genome. The presence of this gene only in the genome of *Er*. *dacicola* and not the genomes of free living *Erwinia* or *Enterobacter* sp. OLF, suggests that it may serve an important role in the association with the olive fly and warrants further investigation. Endosymbionts that detoxify the phenolics may have allowed the tephritid ancestor of the olive fly to be able to feed on olives. Additionally, these phenolics may also reduce microbial competition for the olive fly gut niche. While other bacteria are found in the olive fly gut, they are usually present in lower abundance or in the absence of *Er*. *dacicola*. Perhaps phenolic catabolism in the endosymbiont allows the fly to feed on an underused food source, while providing the bacteria a habitat and effective vertical transmission to future hosts. The extent to which hypothetical proteins encoded in the *Enterobacter* sp. OLF and/or *Er*. *dacicola* genomes serve to degrade olive phenolics remains to be investigated.

### *Erwinia* sp. – plant pathogens and insect mutualists?

Many of the described *Erwinia* (including those formerly belonging in the *Erwinia herbicola-Enterobacter agglomerans* complex) are insect-associated and their relationship with their host(s) varies. *Er*. *dacicola* is an obligately host-associated endosymbiont that has only been found in, and associated with, the olive fly. Whereas, *Pantoea (Erwinia) stewartii* is a plant pathogen harbored by flea beetles during winter and transferred between plants during the growing season by the insect vector^50^. *Erwinia* species also include free-living phytopathogens, such as *Er*. *amylovora* and *Er*. *pyrifoliae*, that may be transferred between plants by pollinators in addition to dispersal by wind and water-dependent mechanisms^51–53^. Many of the described *Erwinia* spp. also vary along a continuum in terms of their microbe-plant interactions. *Er*. *amylovora*, *Er*. *pyrifoliae*, *Erwinia* Ejp617, and *P*. *stewartii* can survive within a diversity of plant tissues and cause serious and costly crop diseases such as fire blight and Stewart’s wilt^51–53^. In contrast *Er*. *tasmaniensis* and *Er*. *billingae* exist as epiphytes or saprophytes on plant surfaces or within necrotic tissue, but cause no plant disease symptoms^53^. Increasingly, non-phytopathogenic, potentially plant- or insect-beneficial *Erwinia* species are being identified. However the potential benefit the *Erwinia* spp. has for the insect host, if any, has been shown to vary within a host: for example one *Erwinia* sp. was found to be beneficial to thrips feeding on cucumber leaves, but detrimental when they fed on cucumber leaves and pollen^54^.

To our knowledge, few studies have examined the potential benefits that insect vectors may receive from the plant pathogenic *Erwinia* species they transport. All *Erwinia* spp. that we examined synthesized vitamins important for basic insect metabolism. Based on the presence of multiple vitamin synthesis pathways in the olive fly endosymbiont, *Er*. *dacicola*, and the other *Erwinia* spp. examined, we hypothesize that insect hosts may obtain vitamins from these vectored plant-pathogenic bacteria. In this way, all *Erwinia* spp. may provide benefit to the insect hosts associated with their transport to different plants.

*Er*. *dacicola* is most closely related to the plant pathogens *Er*. *persicina* and *Er*. *amylovora*^10^. Although DNA from *Er*. *dacicola* is found in the feeding tunnels that the larvae make through the olives^10^, the *Er*. *dacicola* genome lacks genes for cellulases, pectinases, or exoenzymes used by the “soft rotting *Erwinia*/*Pectobacterium*” for establishing infection and necrosis. The observation that tunnels in the olives are restricted to the immediate area on which the larvae feeds and do not expand into the rest of the fruit, suggests that the tunnels are created mechanically by larval feeding and are not enhanced by the introduction of cellulolytic or pectolytic enzymes. However, proteases commonly found in plant pathogenic relatives were present in the *Er*. *dacicola* genome. Since the larvae feed on the olive fruit, the flies seem to have little impact on the olive tree itself. Olive flies do not damage the seed and it is questionable whether the damage they cause to the fruit limits seed dispersal or germination.

## Conclusions

The genomes of *Er*. *dacicola* and *Enterobacter* sp. OLF suggest that neither of these bacteria can fully supplement the diet of the olive fly across development. Both bacteria may degrade different secondary compounds of the olive making the olive fruit more palatable to larvae. Unlike some insect-associated endosymbionts, both *Er*. *dacicola* and *Enterobacter* have genomes that are similar in size and %GC content to their free-living relatives.

There has been much debate as to the composition of the olive fly microbiome. *Er*. *dacicola* seems to be considered a dominant, or perhaps primary endosymbiont^3,4,6,10,13,14^. However, other bacteria, including *Enterobacter* sp. OLF have been found in an assortment of wild and laboratory fly populations at different densities^3,9,10^. *Enterobacter* sp. OLF can survive in all life stages of the olive fly and in the ovipositor of a female^10^, though it seems often to be present at lower densities than *Er*. *dacicola*. Since the *Enterobacter* sp. OLF genome includes an overlapping gene set to that of *Er*. *dacicola*, it may be able to at least partially functionally substitute for *Er*. *dacicola* acting as a probiotic to supplement laboratory olive fly colonies used for sterile insect technique where *Er*. *dacicola* has been lost.

## Methods

### *Enterobacter* sp. OLF sample preparation

*Enterobacter sp*. *OLF* was isolated on LB from a wild male olive fly (*Bacterocera oleae*) from the University of Arizona population. The male olive fly was surface-sterilized in 1% sodium hypochlorite and 0.1% Triton-X 100 as described previously^10^. The abdomen was cut from the thorax under a laminar flow hood using sterile forceps and scalpel. The abdomen was then ground in homogenization buffer^55^ using a sterile plastic pestle in a 1.5 ml sterile microfuge tube, diluted in homogenization buffer to 1 × 10^−8^, and serially diluted on LB. Plates were incubated at 28 °C for 48 h. All colonies were of a smooth, round, white morphology and isolates were recovered by streaking for single colonies on LB at 28 °C for 24 h. A single colony was picked from the restreaked plate and grown in LB, shaking, at 37 °C overnight. A 40% glycerol stock was made of the culture and stored at −80 °C. DNA was extracted from the remainder of the overnight culture using the Gram-negative protocol in the DNeasy kit (Qiagen, Valencia, CA). A ~1500 bp product was amplified from the extracted DNA using the universal 16 S rRNA primer set 10 F and 1507 R. The sequenced PCR amplicon had a 100% BLASTN match to isolate *Enterobacter sp*. i1 (Accession no. GQ478379)^10^.

### *Er*. *dacicola* sample preparation

Since *Er*. *dacicola* cannot be cultured, whole genome amplification was conducted from the DNA of multiple pooled esophageal bulbs from insects collected in Orville, CA, USA. In a laminar flow hood, four sets of 6–10 esophageal bulbs from ~1 month old surface sterilized olive flies were dissected into homogenization buffer^55^. These pools of 4 sets of esophageal bulbs were rinsed in homogenization buffer and kept on ice or in the fridge. The four samples were then centrifuged separately at 3000 × g for 2 min, resuspended in 100 µl fresh homogenization buffer, and homogenized with a 1.5 ml sterile hand-held pestle. The homogenate was centrifuged at 500 × g for 2 min to pellet insect tissues. The supernatant was removed to a fresh microfuge tube and centrifuged at 2000 × g for 2 min to pellet bacteria. At all centrifugation steps, the samples were checked under the microscope for contamination with host tissues. The bacterial pellet was resuspended in 180 µl ATL buffer and the DNA was extracted using the Gram-negative protocol in the DNeasy kit (Qiagen, Valencia, CA). A ~1500 bp product was amplified from each sample using the universal 16 S rRNA primer set 10 F and 1507 R. The amplicons were digested using *PstI* to confirm a banding pattern distinctive of *Er*. *dacicola*^6^. The amplicon was transformed into *E*. *coli* using the TOPO TA Cloning Kit. For each sample, three colonies were picked for sequencing. Using BLASTN, only one clone of the 12 sequenced had a similarity to *Enterobacter* instead of a 100% similarity to *Er*. *dacicola* GenBank accession number GQ478373. The pooled esophageal bulb sample with the *Enterobacter* clone was discarded. A whole genome amplification was performed using the Illustra GenomePhi V2 DNA amplification kit (GE Healthcare) on the remaining nine clones, e.g. from the three samples where all cloned products had a 100% match to *Er*. *dacicola* using BLASTN. A 16 S rRNA PCR product from the whole genome amplification was digested using *PstI* digest and produced a restriction pattern similar to that of previously sequenced *Er*. *dacicola* 16 S rRNA. Whole genome 16 S rRNA amplicons were cloned, sequenced, and had 100% similarity using BLASTN to *Er*. *dacicola*. *Er*. *dacicola* has been previously categorized into two haplotypes, htA and htB^7^. Sequence data indicated that all of samples contained only *Er*. *dacicola* htB, consistent with previous sequencing of olive flies from the Southwestern United States^10^. All nine whole genome amplification samples were pooled and purified using the QIAquick PCR clean-up kit (Qiagen, Valencia, CA).

### Sequencing and assembly

One sample for each bacterial genome was submitted to the Arizona Genomics Institute at the University of Arizona for library preparation and sequencing. For each genome, ~8 µg of DNA was used to make the library. Each bacterial genome was tagged separately and multiplexed for sequencing on one lane of an Illumina Genome Analyzer II.

For the *Enterobacter* sp. OLF genomes, a total of 28,937,299 paired 75 bp reads were generated and quality trimmed. Sequences were assembled into scaffolds using *de novo* assembly in ABySS-pe (version 1.2.5) with 4 different kmer sizes (40, 45, 50, and 55). A cutoff of 300 bp was applied. The K50 assembly showed the genome size closest to that of the closest relative, *Enterobacter cloaeae* ssp. *cloacae* ATCC 13047. The k50 produced 67 scaffolds with an N50 of 180,401.

For the *Er*. *dacicola* genome, a total of 25,876,346 paired 75 bp reads were generated and quality trimmed. Sequences were assembled into scaffolds using *de novo* assembly in ABySS-pe (version 1.2.5) with 4 different kmer sizes (40, 45, 50, 55). A cutoff of 300 bp was set as the minimum scaffold length. The K55 assembly showed the genome size closest to that the genome, *Er*. *amylovora*. A total of 1,036 scaffolds were produced with a N50 of 5,083 bp.

### Annotation

BLAST databases of the *Enterobacter sp*. *OLF* and *Erwinia* genomes sequenced as of March 20, 2011 were created using a customized Perl script (see Table 2 for genomes used). The potential *Er*. *dacicola* scaffolds were searched against the *Enterobacter* and *Erwinia* BLAST databases. Scaffolds with a 100% match to the *Enterobacter* sp. OLF were discarded. Fasta files of all other potential *Erwinia* scaffolds and the *Enterobacter* sp. OLF scaffolds sequenced from the *Enterobacter* cultured isolate were submitted to the IGS Annotation Engine^56,57^, using Glimmer to identify ORFs.

### Data Deposition

The sequenced reads of Enterobacter sp. OLF and Er. dacicola are available in GenBank. as LJAN00000000 and LJAM00000000, respectively. All scaffolds with more than 10 Ns were split into contigs at the site of the Ns by NCBI.

### Sequencing depth

The reads in FASTQ files were aligned to the assembled genome using BWA (v. 0.7.6a)^58^. Sequencing depth was calculated with MPILEUP implemented in SAMTOOLS (v. 1.4)^59^.

### COGs

A BLASTN search of the genomes of each of the olive fly endosymbionts and their free-living relatives was run against the 2014 COGS database (ftp://ftp.ncbi.nih.gov/pub/COG/COG2014/data). Each gene was associated with one or more COG categories based on a BLASTN e-value cutoff of 10^−20^. Pearson Chi-Squared approximate multinomial exact tests evaluated the distributions of loci into the different COG categories having >5 loci in the category by comparing *Enterobacter* sp. OLF to the COG distribution of other sequenced *Enterobacter* (Table 2) and by comparing *Er*. *dacicola* to the COG distribution of the other sequenced *Erwinia* (Table 2).

### Genome comparison and content

Sybil^60^ was used to generate Jaccard clusters of orthologs (JOCs) for *Er*. *dacicola* using bi-directional best BLAST matches while MUGSY^61^ and MUGSYAnnotator^31^ were used to predict COGs for *Enterobacter* sp. OLF. Pathway Tools^62^ was used to predict the metabolic pathways.

## Electronic supplementary material

Supplementary Information

Table S1

Table S2

Table S3

Supplementary Data File 1

Supplementary Data File 2
