# Supplementary material for: Comparative genomics of the Erwinia and Enterobacter olive fly endosymbionts
Source: Sci Rep. 2018 Oct 29;8:15936. doi: 10.1038/s41598-018-33809-w (PMC6205999; doi:10.1038/s41598-018-33809-w)
Supplement: Supplementary file 1 — Supplementary Information [file 41598_2018_33809_MOESM1_ESM.docx]

**Comparative genomics of the *Erwinia* and *Enterobacter* olive fly endosymbionts**

Anne M. Estes^1,*,†^, David J. Hearn^2^, Sonia Agrawal^1^, Elizabeth A. Pierson^3^, Julie C. Dunning Hotopp^1,4,*^

## Supplementary Files

###
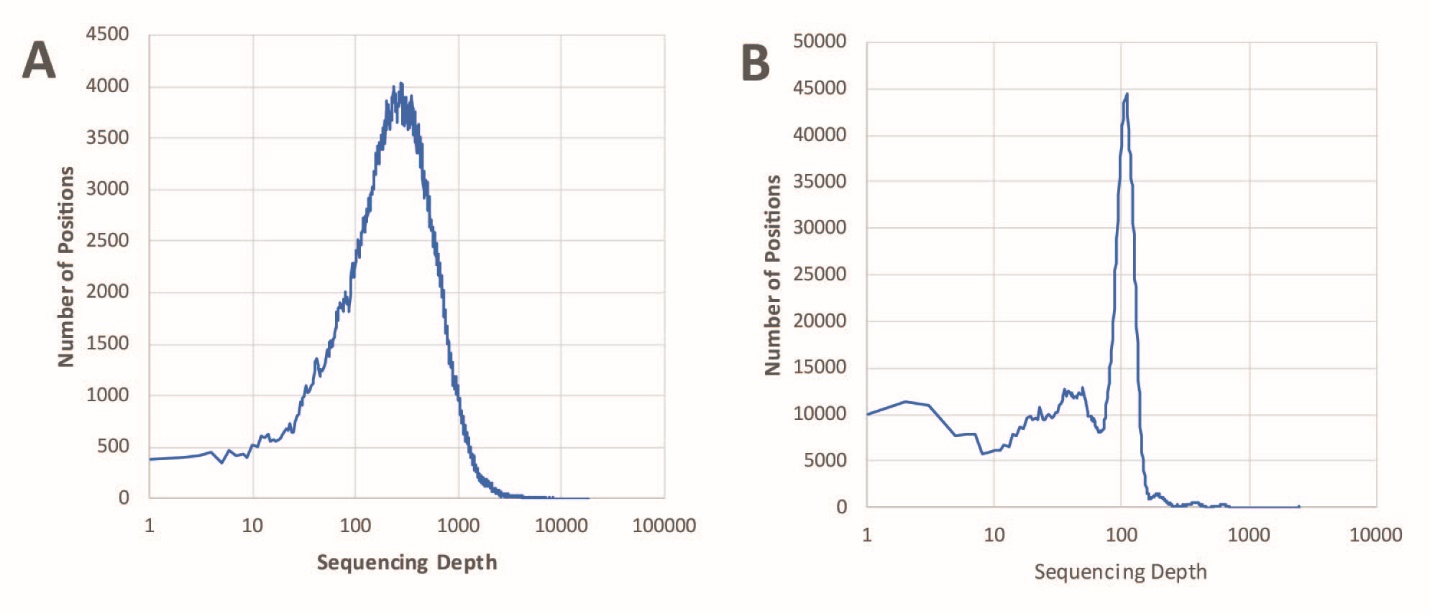
Supplementary Fig S1. Frequency distribution of sequencing depth

To compare reads from two independent sequence analyses of *Er. dacicola*, sequencing reads generated in the US as well as those generated in Liverpool were aligned to the larger US genome assembly with BWA ALN and BWA SAMPE with duplicates removed with Picard, and the sequencing depth measured at each position in the genome with MPILEUP in SAMTOOLS. The frequency distribution of the sequencing depth per base pair is illustrated for (A) the US reads and (B) the run of Liverpool reads with the highest sequencing depth. For US reads, the sequencing depth distribution across these scaffolds was unimodal, but asymmetric, with a mean of 645X sequencing depth, a median of 476X sequencing depth, and a mode of 280X sequencing depth. For the Liverpool reads, the sequencing depth distribution across these scaffolds was largely unimodal and symmetric, with a mean of 107X sequencing depth, a median of 102X sequencing depth, and a mode of 109X sequencing depth. A visual examination of the frequency distribution of the sequencing depth of the Liverpool data reveals five local modes at 2x, 50x, 200x, 400x, and 700x.

### Supplementary Table S1. Shared core clusters *Er. dacicola*

Excel workbook with sheets for the lists of shared core clusters of non-duplicated genes as determined by MUGSY that were used for the Venn Diagrams in **Figure 5**.

### Supplementary Table S2. Shared core clusters *Enterobacter* sp. OLF

Excel workbook with sheets for the lists of shared core clusters of non-duplicated genes as determined by JOCs that were used for the Venn Diagrams in **Figure 4**.

### Supplementary Table S3. Summary of Results with Pathway Tools

### Supplementary Data File S1. Scaffolds and genes with above average and below average sequencing depth

Excel worksheets containing scaffolds and genes with above average and below average sequencing depth.

### Supplementary Data File S2. COG gene frequencies

Excel worksheets containing COG gene frequencies.
